# Preventive Moderate Continuous Running-Exercise Conditioning Improves the Healing of Non-Critical Size Bone Defects in Male Wistar Rats: A Pilot Study Using µCT

**DOI:** 10.3390/life10120308

**Published:** 2020-11-24

**Authors:** Céline Bourzac, Morad Bensidhoum, Mathieu Manassero, Christine Chappard, Nicolas Michoux, Stéphane Pallu, Hugues Portier

**Affiliations:** 1 B3OA, UMR CNRS 7052, INSERM U1271, Université de Paris, 75010 Paris, France; c_bourzac@yahoo.fr (C.B.); morad.bensidhoum@paris7.jussieu.fr (M.B.); mathieu.manassero@vet-alfort.fr (M.M.); christine.chappard@inserm.fr (C.C.); stephane.pallu@univ-orleans.fr (S.P.); 2 Département Elevage et Pathologie des Equidés et des Carnivores, Ecole Nationale Vétérinaire d’Alfort, 94700 Maisons-Alfort, France; 3 Département de Radiologie, Institut de Recherche Expérimentale et Clinique, Cliniques Universitaires Saint-Luc, Université Catholique de Louvain, 1200 Brussels, Belgium; nicolas.michoux@uclouvain.be; 4 Collegium Science & Technique, 2 Allée du Château, Université d’Orléans, 45100 Orléans, France

**Keywords:** physical exercise, continuous running training, bone repair, non critical-size bone defect, rats

## Abstract

Although physical exercise has unquestionable benefits on bone health, its effects on bone healing have been poorly investigated. This study evaluated the effects of preemptive moderate continuous running on the healing of non-critical sized bone defects in rats by µCT. We hypothesized that a preemptive running exercise would quicken bone healing. Twenty 5-week-old, male, Wistar rats were randomly allocated to one of the following groups (*n* = 10): sedentary control (SED) or continuous running (EX, 45 min/d, 5 d/week at moderate speed, for 8 consecutive weeks). A 2 mm diameter bone defect was then performed in the right tibia and femur. No exercise was performed during a 4 week-convalescence. Healing-tissue trabecular microarchitectural parameters were assessed once a week for 4 weeks using µCT and plasma bone turnover markers measured at the end of the study protocol (time point T12). At T12, bone volume fraction (BV/TV; BV: bone volume, TV: tissue volume) of the healing tissue in tibiae and femurs from EX rats was higher compared to that in SED rats (*p* = 0.001). BV/TV in EX rats was also higher in tibiae than in femurs (*p* < 0.01). The bone mineral density of the healing tissue in femurs from EX rats was higher compared to that in femurs from SED rats (*p* < 0.03). N-terminal telopeptide of collagen type I in EX rats was decreased compared to SED rats (*p* < 0.05), while no differences were observed for alkaline phosphatase and parathyroid hormone. The study provides evidence that preemptive moderate continuous running improves the healing of non-critical sized bone defects in male Wistar rats.

## 1. Introduction

Physical exercise (PE) has unquestionable beneficial effects on bone health [1,2,3,4,5]. Using rats as a model, the effects of different exercise modalities on bone biochemical characteristics and biomechanical properties have been previously investigated [6,7,8]. Among PE modalities, treadmill running is one of the most common weight-bearing exercises in rats. On normal bone, literature reports provide the following evidence. Moderate continuous running (MCR) on a treadmill, (i) increased the bone mineral density (BMD) in the tibiae or femora as a result of increased trabecular volume, number or thickness of trabeculae, (ii) decreased trabecular separation [6,9,10,11,12,13] and (iii) increased cortical bone volume in the tibiae [11], compared to respective results obtained in sedentary controls. Treadmill MCR also increased calcium accumulation in the bone, stimulating bone formation and suppressing bone resorption in young female rats [14]. Of note, some prophylactic effects of MCR on bone health were reported. MCR thus prevented trabecular bone loss related to hormone suppression in ovariectomized female [11] or castrated male [15] rats. MCR also prevented decreased femoral BMD induced by age, most likely by limiting bone resorption [16]. Finally, treadmill exercise prevented deterioration of subchondral bone in a rat model of osteoarthritis [17].

The positive effects of PE have also been investigated on the repair of various tissues (including bone) in rats. In myocardial tissue treadmill running for 12 weeks (initiated 24 h after experimentally-induced infarction) showed positive effects on cardiac remodeling and function, resulting in an increased left ventricle free wall in systole or in an improved ejection fraction [18]. In cartilage, regular treadmill running improved Wakitani cartilage repair scores (in osteochondral defect in the femoral groove after 8 weeks) compared to sedentary rats [19]. In bone tissue, swimming (with a load of 5% body weight) improved osteotomy healing at the end of bone consolidation [20]. Compared to swimming, running exercise (intensity not given) starting 24 h after a non-critical-sized bone defect surgery, improved trabecular radio-opacity after 14 days of exercise [21]. In a model of bone defect treated with biodegradable cement, treadmill exercise for 30 consecutive days (starting 7 days postoperatively) improved bone repair in terms of, (i) defect filling (decreased residual defect areas and increased speed of repair) and (ii) repaired tissue strength compared to sedentary controls [22].

Although the benefits of PE on bone health have been widely documented [6,9,10,11,12,13,23,24], most of the studies investigating the effects of PE on bone repair have focused on PE a posteriori, i.e., after an injury was sustained. Two studies addressed the effects of previous exercise training on subsequent bone healing by evaluating the effects of moderate continuous running (MCR) on the healing of a manually-induced tibial fracture in mice [25] or non-critical-sized defect in the tibia in rats [26]. Increased rates of collagen synthesis and higher levels of calcium in the callus from mice subjected to exercise compared to sedentary mice [25], and increased bone formation rate, type I collagen and osteocalcin expression [26] suggested enhanced bone healing with prior exercise. These studies, however, were limited to the tibial bone and to chemical or immunohistochemical evaluation of the healing tissue. Moreover, the follow-up evaluation of the healing tissue was restricted to 1 or 2 time-points within 14 days maximum. Finally, BMD imaging assessment was performed in the whole tibia, not the defect only [26]. The effects of PE on the femur or on the systemic bone turnover markers and healing bone tissue imaging and longitudinal follow-up, were not investigated.

Thus, preemptive PE may have prophylactic effects on bone healing, but studies are limited both in the number of parameters assessed and in time of follow-up. To address this gap in knowledge, the aims of this study were: (i) to follow the evolution of the healing tissue microarchitecture using µCT over a period of 4 weeks, (ii) assess the BMD in the healing tissue specifically and (iii) evaluate systemic bone turn over markers after 8 weeks of preemptive MCR exercise in male Wistar rats. It was hypothesized that previous MCR exercise improves the healing of non-critical-sized bone defects compared to sedentary lifestyle, both in the tibia and femur.

## 2. Materials and Methods

### 2.1. Animals

Twenty 5-week-old, male, Wistar rats, weighing 201 ± 10 g, were purchased from Elevage Janvier (Le Genet-St-Isle, France), acclimated for 1 week to the new facilities and for 1 week to the treadmill. Rats were then randomly assigned to one of the 2 following groups (*n* = 10 each): sedentary control (SED) or moderate continuous running exercise (EX). The rats were housed all along the experiment in controlled facilities (4 per standard cage, under a 12 h light/dark cycle, kept at a constant temperature of 21 ± 2 °C). A commercial standard diet (Genestil, Royaucourt, France) and tap water were provided ad libitum to all animals. Asides from free movement in their cages, all the rats had no access to any other activity such as wheel running. Moreover, during the recovery period, no rats showed signs of discomfort or lameness of the operated limb.

The experimental protocol was approved by the Ethics Committee on Animal Research of Lariboisiere/Villemin (Paris, France) and from the French Ministry of Agriculture (Paris, France; APAFIS # 9505), and was carried out in accordance with the European Guidelines for Care and Use of Laboratory Animals (Directive 2010/63/EU).

### 2.2. Experimental Design

**Figure 1:**
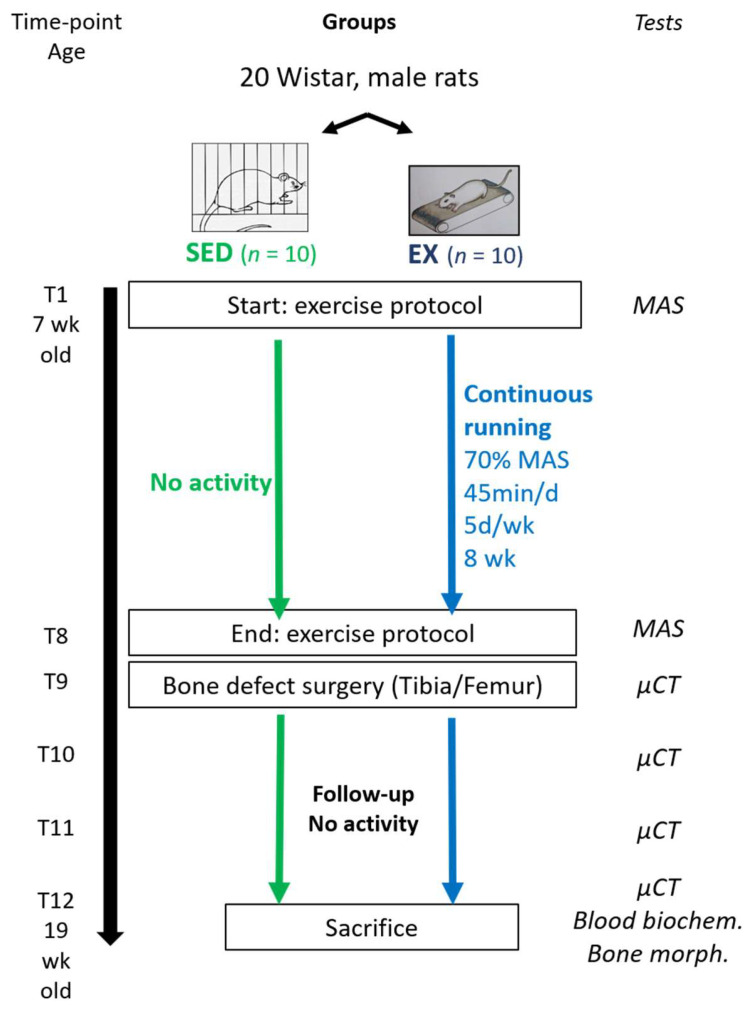
Study design SED: sedentary control, EX: moderate continuous treadmill running exercise, MAS: maximal aerobic speed, µCT: micro-computed tomography, biochem.: biochemistry analysis, morph.: morphometry.

The study was divided in two phases (Figure 1). The first phase involved treadmill running exercise for 8 consecutive weeks (from time-point T1 to T8). The second phase involved a non-critical sized bone-defect surgery, followed by 4 weeks of follow-up (from time-point T9 to T12). Femur and tibia were chosen because they are load-bearing bones that may be mechanically impacted by treadmill training program.

At the end of the follow-up period (T12), all rats were sacrificed by cardiac puncture and exsanguination, after induction of general anesthesia with 5% isoflurane in air. Blood was collected on heparinized tubes, immediately cooled, centrifuged at 700× *g* at 4 °C for 10 min and plasma stored frozen at −80 °C until assays were performed.

### 2.3. Body Parameters, Food and Water Follow-Up

Quantities of ingested chow and water were recorded weekly during the training period. Body weights were recorded at 5 (upon arrival, time-point T0), 15 (T8) and 19 (T12) weeks of age, body lengths (nose to anus) at T0 and T12, and body mass index (BMI) calculated as body weight (g)/length^2^ (cm^2^) at T0 and T12 [27].

### 2.4. Exercise Training Protocols

Prior to the beginning of the exercise protocols, the rats were placed on a motor-driven treadmill [28] for adaptation for 4 days. On the 5th day, the maximal aerobic speed (MAS) was determined for each rat, using a progressive running test (adapted from Krzesiak et al., 2019 [29]), starting with warming at 10° inclination and a speed of 13 m/min for 5 min, then increments of 4 m/min every 2 min until 17 min were reached, then increments of 4 min/min every 1 min 30 s. The test was conducted until fatigue occurs. Fatigue was defined as when the rats could no longer keep pace with the treadmill speed, despite 2 consecutive stimulations with air-compressed sprays. The test was then stopped and the last fully sustained increment speed defined as the MAS for the rat. Since speed could not be adapted within each treadmill lane, groups had to be formed for training and the MAS for each group corresponded to the lowest MAS within the group. At the beginning of the exercise protocol (time-point T1), the rats were 7 weeks old.

The EX group performed MCR activity on the treadmill, 0° inclination, 45 min per day, 5 days per week, for 8 consecutive weeks, starting with a 5 min warming session at the speed of 12 m/min followed by 40 min corresponding to 70% of MAS (i.e., 15 m/min). Exercise stimulation was provided, when needed, with air-compressed sprays. Electric shocks were not used at any stage. The SED group did not perform any daily physical activity beyond normal movements in their cages. A MAS test was conducted at the end of the exercise protocol (T8) to validate exercise conditioning [30].

### 2.5. Non-Critical Sized Bone Defect Surgery

#### 2.5.1. Anesthesia

Prior to surgery, the rats were anesthetized in an induction box with 5% isoflurane (Forene^®^) in oxygen, then transferred to a mask for anesthesia maintenance with 3% isoflurane in oxygen. A stable body temperature was maintained using a heating device (maintained at 37 °C). The rats were then premedicated with a single dose of enrofloxacin (10 mg/kg, subcutaneously (SC), Baytril^®^ 5%) for antimicrobial prophylaxis, and buprenorphine (0.2 mg/kg, SC, Buprecare^®^ 0.3 mg/mL) for analgesia. The right hind limb was clipped, aseptically prepared and draped.

#### 2.5.2. Surgical Procedure

**Figure 2:**
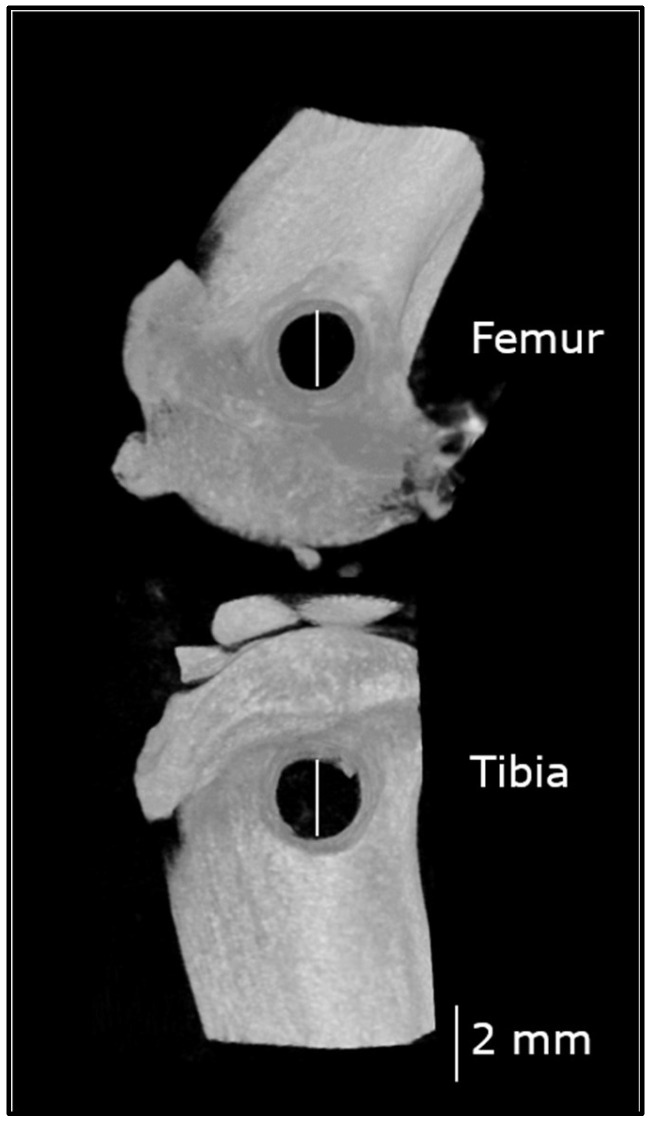
Non critical-sized bone defects in the tibia and the femur immediately after surgery. These microcomputed tomography (µCT) images display the 2 mm bone defects in the distal metaphysis of the femur and the proximal metaphysis of the tibia. This figure is a composite image of reconstructions obtained from the femur and the tibia, separately.

A parapatellar approach was performed on the lateral aspect of the limb, with skin incision centered over the femoro-patellar joint. The fascia lata was incised and muscles were retracted caudally. A cylindrical defect was created using a 2 mm drill bit and a ultraviolet-sterilized, surgery-dedicated drill (Dremel^®^ 4300, Robert Bosch France, Saint-Ouen, France), in the distal femoral metaphysis, at the level of the lateral collateral ligament insertion, between the lateral collateral ligament and the insertion of the extensor digitorum longus muscle, and in the proximal tibial metaphysis, at the level of the collateral ligament insertion, between the lateral collateral ligament and the patellar tendon. Irrigation with saline was provided throughout the drilling procedure to avoid thermal necrosis. The drilled holes were then rinsed to discard any bone fragments (Figure 2). The fascia lata, subcutaneous tissue and skin were closed in 3 plans using an absorbable monofilament (Biosyn^®^ 5-0). The wound was irrigated with lidocaine (Lurocaïne^®^, 20 mg/mL) between each plan to provide additional analgesia. Buprenorphine (0.1 mg/kg, SC) was provided 12 h after surgery.

All rats recovered successfully from surgery. They did not display any discomfort or lameness in their operated limb from the day after surgery to the day they were euthanized. The day after surgery, however, 1 rat of the SED group presented with wound dehiscence. The wound was debrided and sutured close without any further complications.

### 2.6. Postoperative Microcomputed Tomography (µCT) and Computation

Anesthesia was induced and maintained as described above. The right distal femur and proximal tibia were imaged during the same procedure using a high-resolution µCT (Skyscan 1176, Bruker, Belgium) once a week following surgery, for 4 weeks (time-points T9, T10, T11 and T12). These time-points were chosen on the basis that a 2 mm × 6 mm cortical defect in rat femurs filled within 40 days [31].

#### 2.6.1. µCT Settings

The µCT images were obtained at the following setting: source voltage 50 kV, source current 475 µA, matrix 1000 × 668, exposure time 40 ms, rotation step 0.7-degree, frame averaging 5, filter aluminum 0.5 mm thick and pixel size 35.6 µm.

#### 2.6.2. µCT Reconstruction and Data Computation

Images were reconstructed (separating the femur from the tibia) using NRecon software, 16 bits (v1.7.0.4, Skyscan). For the quantitative analysis of bone formation within the femoral and tibial bone defects, all µCT reconstructions were oriented using DataViewer software (v1.5.2.4, Skyscan, Aartselaar, Belgium), so that the bone defect appeared as a circle on each consecutive slice. For bone volume computation, images were converted to 8 bits and a cylindrical volume of interest (VOI; diameter 2.3 mm, so that the size was close to, but not including the rim of the drilled hole), centered over the bone defect was defined within the cancellous bone, as previously described for the cortical bone [32,33]. The length of the cylinder represented the centrally located 75% of the total length of the defect at each time point. This methodology was used to evaluate the exact same region at each time point, accounting for the radial bone growth and differences in defect length, due to variations in bone defect location with respect to the physis. The bone volume fraction (BV/TV, a measure of part of cancellous space filled with trabeculae [34]; BV: bone volume, TV: tissue volume), specific bone surface (BS/BV; BS: bone surface), structure model index (SMI) and trabecular pattern factor (Tb.Pf) were computed from these VOI within bone-specific threshold (40–160 gray values) at each time point (T9 to T12). Bone mineral density (BMD) was computed from these VOI at T12 only, using hydroxyapatite phantoms for calibration, with CTAn software (v1.16.4.1, Skyscan, Aartselaar, Belgium) [35].

### 2.7. Blood Analyses

Total alkaline phosphatase (ALP), a marker of bone formation, was assayed using a standard laboratory analyzer (Selectra xl, Elitech) and enzymatic kinetic kit (ALP (DEA) SL, Elitech), in the same run for all the samples, following the manufacturers’ instructions. The limit of detection was equal to 6 U/L.

Cross linked N-Telopeptide of type I collagen (NTx), a marker of bone resorption, and intact parathyroid hormone (PTH), a key regulator of the phospho-calcic metabolism, were measured using established rat enzyme linked immunosorbent assay kits (Antibodies-online GmbH, Aachen, Germany [36], and Immutopics, Inc., San Clemente, CA, USA [37], respectively), following the manufacturers’ instructions. The intra-/interassay coefficients of variation and limit of detection for NTx were <12% and 2.47 ng/mL respectively. The intra-/interassay coefficients of variation and limit of detection for PTH were <8.9% and 3 pg/mL respectively.

### 2.8. Data Analysis and Statistical Analysis

Statistical analyses were performed using a commercially available software (StatView, Version 5.0.; SAS Institute Inc., Cary, NC, USA). Descriptive statistics were reported as means ± standard deviation of the mean (SD) or median (with 25th and 75th percentiles) according to the normality of the data distribution (Shapiro–Wilk test). Differences between EX and SED groups in terms of body characteristics, MAS, blood analysis and BMD were then assessed using a Mann–Whitney U test. The level of significance was set at *p* < 0.05.

Evolution over time of parameters BV/TV, BS/BV, SMI and Tb.Pf was first studied using boxplots. Then, a four-way ANOVA was performed for each parameter of interest to assess potential differences in measurements between groups, bones and time-points (fixed effects) and between animal (random effects). The function ANOVAN (Matlab software v.R2017a; The MathWorks Inc. Natick, MA, USA) was used for the analysis. The significance level for these tests was *p* < 0.025 (Bonferroni correction). When the analysis of variance demonstrated statistically significant differences between groups means, a pairwise comparison between these groups was performed using the function MULTCOMPARE (Matlab). This function allowed comparing the marginal means from the factors group, bone and time points in averaging out the effects from the factor animal [38]. Due to the multiple comparisons that were performed, a Tukey–Kramer correction was applied to this test.

## 3. Results

### 3.1. Body Parameters, Food and Water Consumption

Results are summarized in Table 1. At the beginning of the protocol, body mass indexes were not statistically different between SED and EX groups. Body mass indexes and weight gains were not statistically different between SED and EX rats throughout the study time. Food and water consumptions were not different between SED and EX rats (food: 25.1 ± 4.1 and 27.6 ± 2.9 g, for SED and EX rats respectively; water: 31.4 ± 3.1 and 37.6 ± 11.3 mL respectively).

### 3.2. Exercise Training

At T1, the MAS was not significantly different between EX and SED rats (Table 1). At T8, the MAS was higher in EX rats compared to that in SED rats (MAS^EX^: 37.3 ± 7.4 m/min, MAS^SED^: 18.3 ± 5.4 m/min, *p* < 0.01). The MAS improved (*p* < 0.05) by 8.1 m/min in the EX rats between T1 and T8, validating exercise conditioning.

### 3.3. Microcomputed Tomography Bone Formation and Bone Mineral Density Assessment

**Figure 3:**
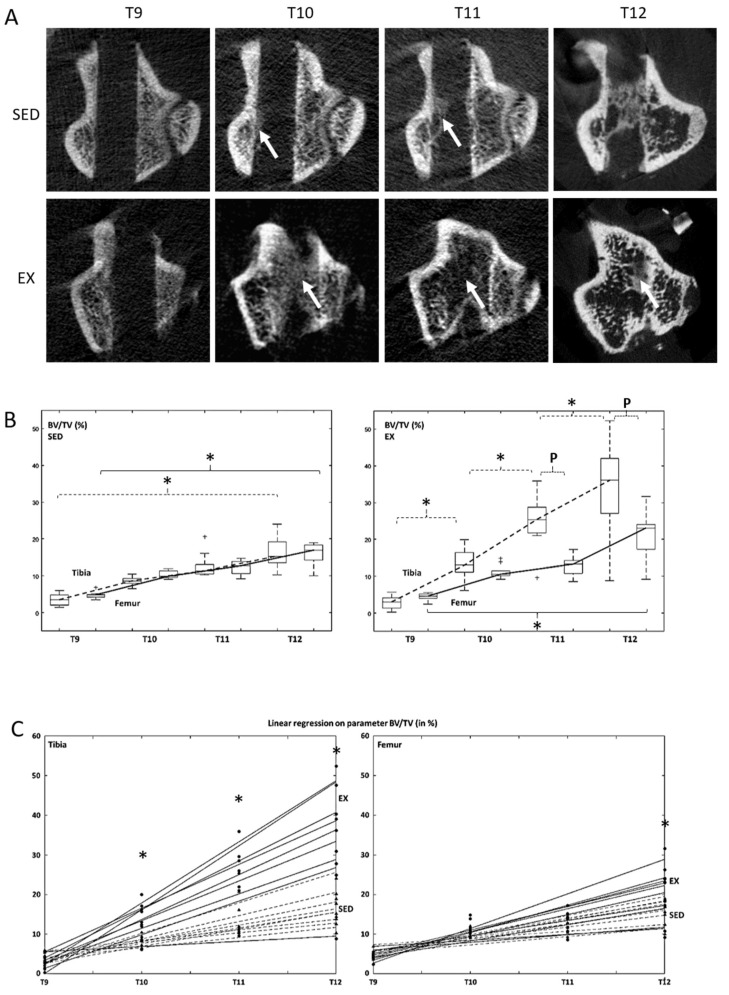
The healing of a non-critical sized bone defect in the tibia and the femur of sedentary (SED) and running (EX) male Wistar rats was followed for 4 weeks. (**A**) Representative images of progressive bone healing in the tibia of SED and EX rats over time (transverse views through the central portion of the defect). White arrows indicate healing tissue. The defect is almost completely filled-in in EX rats at the 4 week follow up. (**B**) Evolution of parameter BV/TV (in %) in tibia and femur rats over time is displayed as a boxplot for both SED and EX rats. On each box, the central mark indicates the median, and the bottom and top edges of the box indicate the 25th and 75th percentiles. The whiskers extend to the most extreme data points not considered outliers (‘+’). Similar kinetics are observed in SED rats, regardless of the bone, while a steeper slope in the tibia compared to that of the femur is observed in EX rats. Four-way ANOVA, *n* = 9 in each group. * indicates a statistically (*p* < 0.05) significant difference between time-points and P indicates a statistically significant difference between bones. (**C**) Evolution of parameter BV/TV (in %) in SED and EX rats over time is displayed as a boxplot for both tibia and femur. Kinetics show a steeper slope in the tibia from EX rats compared to respective results from SED rats and similar slopes in the femur, regardless of the group. Four-way ANOVA, *n* = 9 in each group. * indicates statistically significant differences between SED and Ex rats.

Representative images of bone defect healing over time are given in Figure 3A. Between T9 and T12, BV/TV increased in both SED and EX rats and for both tibias and femurs (Table 2, BV/TV^SED^: *p* < 0.0001 for tibias and *p* = 0.006 for femurs; BV/TV^EX^: *p* < 0.0001 for tibias and *p* < 0.0001 for femurs). In the tibias, BV/TV in EX rats were higher (*p* = 0.0012) at T10, T11 and T12, than respective results obtained in SED rats. In the femurs, BV/TV in EX rats was higher (*p* < 0.0001) than respective results obtained in SED rats, at T12 only (Figure 3C). In SED rats, a significant increase in BV/TV was observed only between T9 and T12, regardless of the bone.

In contrast, significant increases in BV/TV in EX rats were observed between each time-point for the tibias (*p* < 0.0001), while no significant difference was observed at intermediate time-points for the femurs. Overall, no difference in BV/TV between tibias and femurs was observed in SED rats, regardless of the time-points. In contrast in EX rats, BV/TV was higher in tibias at late time-points (T11 and T12), than respective results obtained in femurs (*p* = 0.01; Figure 3B).

A significant (*p* < 0.0001) time–bone interaction was observed for Tb.Pf, suggesting that the effect of time on Tb.Pf depended on the bone in which it was measured (Table 2). BS/BV (*p* < 0.0001) and SMI (*p* < 0.001) only depended on time. No significant difference with respect to group (SED vs. EX), bone (tibia vs. femur) and animals were observed (Table 2).

At T12, the femoral BMD in EX rats was higher (*p* = 0.03) than respective results in SED rats. No significant difference in tibial BMD was observed between EX and SED rats (Table 2).

### 3.4. Blood Analysis Parameters

At T12, plasmatic NTx was lower (*p* < 0.05) in EX rats compared to respective results obtained in SED rats. Although not statistically significant, plasmatic PTH was higher in EX rats. Plasmatic ALP was not statistically different between SED and EX rats (Table 3).

## 4. Discussion

In this study, we investigated whether exercise in the form of treadmill MCR prior to a bone injury can be a preemptive method that increases bone healing in a non-critical bone defect rat model. Our goals were to determine the effects of preemptive treadmill MCR on (i) healing bone tissue microarchitecture and mineral density in the femur and the tibia and (ii) on systemic bone turn over markers.

### 4.1. Preemptive MCR Improves Bone Healing Tissue Microarchitecture and Mineral Density

Our results demonstrated that bone healing in both the tibiae and femora was significantly improved in running rats compared to sedentary rats. Specifically, BV/TV in the tibia and femur from running rats was higher at the end of the study than respective results obtained from sedentary rats. Moreover, the kinetics of the healing process in the tibia from running rats followed a rather linear progression and were improved at all time-points, compared to sedentary rats. Finally, the BMD of the femoral healing tissue was also improved in running rats compared to sedentary rats. These two results provide evidence that the beneficial effects of MCR on bone healing persist for several weeks, since differences between SED and EX rats increased with time. This is consistent with the previously reported results that the beneficial effects of tibia loading for 3 days per week, for 4 weeks on bone volume fraction in mice persisted 8 weeks after loading cessation [39].

In contrast, preemptive exercise had no effect on BS/BV, SMI and Tb.Pf. These parameters, however, decreased over time, as observed with bone maturation in longitudinal bone repair studies [40,41]. BS/BV is measured based on triangulation of the surface, SMI and Tb.Pf correspond to the surface/volume ratio before and after dilation. In a theoretical point of view, BS/BV, which represents the relative surface to volume, is higher in the case of a dense network of thin structures. This can be observed in newly formed bone such as callus struts compared to mature bone where trabeculae are thicker and clearly designed. SMI close to zero corresponds to ideal plates, 3 to ideal rods and 4 to ideal spheres [42]. Therefore, SMI can provide information about new bone maturity. As previously reported following tooth extraction, the newly-formed bone is more rod-like, as opposed to mature bone, which is more plate-like [41]. Tb.Pf reflects the proportion of concave–convex structures and is an indirect measurement of connectivity with a high proportion of concave structures in the case of highly connected structures. On the contrary, abundant convex structures represent a disconnected trabecular bone network. Lower Tb.Pf indicates a highly connected state of bone structure. To the best of our knowledge, this is the first longitudinal study evaluating the effects of prophylactic exercise on bone healing in young, male, Wistar rats.

### 4.2. Effects of Preemptive MCR on Bone Healing Depend on the Bone Studied

Our results demonstrated that, in the running rats, the kinetics of the healing process in the tibia was steeper, with BV/TV remaining higher at late time-points, than respective results obtained in the femur. In contrast, in the sedentary rats, no such difference was observed. Moreover, the kinetics of the healing process were rather linear in the tibia, while they plateaued between T10 and T11 before increasing again between T11 and T12 in the femur. MCR improved the BMD in the femoral but not the tibial healing tissue. This is in contrast with a previous study, in which the BMD in the tibia was increased following a treadmill running exercise [26]. The BMD, however, was measured in the whole tibia and rather reflects the previously reported effects of exercise on trabecular parameters in the entire bone [6,9,10,11,12,13], than in the healing tissue specifically. Tentative explanations include, but are not limited to, differences in stress intensity or distribution sustained by the femur and the tibia due to the relative orientation of these bones when contacting the ground, differences in bone geometry [11], attenuation of the mechanical stimulus in the femur by menisci or differences in response to running exercise among skeletal sites [12]. Regarding the BMD results, one possible explanation would be that the amount of mineralized tissue within the healing tissue is similar between the tibia and the femur but the distribution of this tissue within the total volume of healing tissue changes as a smaller volume of healing tissue was obtained in the femur [43]. We are not aware of previous studies comparing the healing process within these two bones, and these findings may have interesting implications, such as proper model selection to study bone healing for instance.

In order to explain the effects of MCR on bone healing, we analyzed bone turnover markers in the serum of EX and SED rats. No difference in total ALP, an early marker of osteogenesis, was observed between running and sedentary rats. These results were consistent with previous reports, in which treating osteotomized bone [20] or bone defects [21] with swimming or running exercises did not elicit an increase in ALP. A first hypothesis to that observation is that exercise intensity was not sufficient or too high to increase ALP activity. Data from a recent study [44] using a similar protocol (treadmill running at 16 m/min, 45 min/d and 5 d/week, for 4 consecutive weeks) did not show an increase in serum ALP, whereas running at 12 m/min did. These experiments, however, were conducted in rats with intact bones, limiting the conclusions to healing bones. A second hypothesis is that the size of the bone defect was not sufficient to increase plasma levels of ALP, especially 4 weeks after cessation of the physical activity [21]. In that case, investigating variations in temporal ALP gene expression in the bone tissue during the healing process may provide further information [45].

MCR resulted in a 16% decrease in NTx, compared to sedentary rats. This finding suggests that MCR may favor bone healing and increase BMD by reducing bone resorption, as previously described in healthy bone [46]. This is in contrast with the previous results that MCR (for 4 consecutive weeks) did not induce an increase in osteoprotegerin and tartrate-resistant acid phosphatase in the healing tissue of a non-critical-sized defect [26]. This discrepancy may be explained by the fact that 4 weeks of training were not enough to elicit an effect on bone resorption, while 8 weeks were [46].

PTH is an indirect marker of increased bone remodeling. It has been reported in the literature that PTH regulated bone adaptation to exercise and that running exercise would increase plasma PTH levels [47,48,49]. It has also been demonstrated that the administration of PTH promoted fracture healing [50]. In our study, however, plasma PTH levels in running rats were not significantly different from these in sedentary rats. These results were consistent with a previous study evaluating the effects of exercise on bone remodeling-related hormones, although that study was performed on ovariectomized rats [51]. In [47,48,49], PTH was evaluated at the end of the exercise protocols. Of note in our study, although not statistically significant, PTH levels in running rats were higher than in sedentary rats. Therefore, the effects of running exercise on PTH levels may not have been detectable anymore at the end of a 4 week-convalescence period.

### 4.3. Putative Explanations Pertaining to the Positive Effects of MCR on Bone Healing

Bone healing is a highly orchestrated physiological process, proceeding with a variety of primordial cells, intracellular and extracellular signaling (such as hormones and growth factors for instance [52]), and an osteoconductive matrix, in a defined spatial and temporal sequence [53]. It is now well-established that bone responds to loads [54] and that strains, induced by loading the bone, generate signals, to which cells (osteoblasts, osteoclasts, osteocytes, etc.) may detect or respond [55,56]. PE also has indirect effects on the bone, through bone–muscle interactions: PE induces an increase in muscle mass, which in turn, induces increased strains at the osteotendinous junction. These strains generate an osteogenic stimulus [11], activating the periosteum and enhancing callus formation [57]. Additionally, the effects of PE on mesenchymal stem cells has been reviewed; although little data is available, PE in the form of MCR may enhance bone marrow-derived mesenchymal stromal cell proliferation and osteogenic capacities [58]. Pertaining to bone matrix, literature data is consistent with an increase in collagen type I, the most abundant protein in the bone matrix, with no changes in collagen type III, associated with MCR, pleading for a better bone collagen network organization induced by exercise [26]. Taken together, these results contribute, at least partially, to the positive effects of MCR on bone healing. Noteworthy in our study, food and water consumption and body weight variations were similar between groups. Therefore, these parameters may not explain the differences in healing kinetics observed between sedentary and running rats.

### 4.4. Limitations

We acknowledge that this study presents several limitations. First, it was conducted on still growing male rats in order to observe the effects of PE only, and avoid many physiological interactions (e.g., age: sarcopenia and osteopenia, sex: hormones and possibly osteoporosis). Studies in mice, however, demonstrated that bone responses to mechanical stimuli decrease in adult and aged animals, compared to young ones [59]. Therefore, these results may not be applicable to adult rats or humans. Since bone response to exercise was shown to be sex-dependent [60], conclusions may not be directly drawn for the female population either. Second, moderate continuous running exercise was chosen because it is commonly used and better than low or high intensity running, to increase bone mass in humans and animals [61,62,63]. Further work may include investigations of the effects of different types of running (i.e., continuous vs. intermittent) and different intensities (i.e., low, moderate and high) and different types of exercises (i.e., weight-bearing vs. non weight-bearing, for instance) on bone repair. Finally, the study did not intend to investigate the mechanisms and signaling transduction pathways of bone tissue responses during bone repair associated with MCR. The study aimed at analyzing positive or negative effects of MCR on bone healing. Therefore, the effects of MCR on bone vascularization, calcium intake and to (immuno-) histological and chemical analyses of the healing tissue should be further investigated to explain the observed differences in bone repair between running and sedentary rats [26].

### 4.5. Perspectives in Humans

To the best of our knowledge, the prophylactic effects of running exercise on lower limb bone repair have not been investigated in humans. In fact, studies in humans have mainly focused on rehabilitation after a fracture was sustained and rather investigate resistance exercises, progressive weight-bearing or whole-body vibration. By using still growing rats in our study, these may be a pertinent model to investigate bone repair in young adults, at the bone mass peak [64]. Data retrieved from studies investigated the effects of preemptive exercise on bone healing may provide valuable information to adapt training protocols in servicemen and athletes for instance, populations at increased risks of lower limb fractures [65,66] and shorten the period of convalescence. Further investigation may involve older rats or ovariectomized rats, and different running modalities, to allow inference in the elderly population, at high risk of fracture injuries too [67], and plan personalized preventive exercise strategies.

## 5. Conclusions

In conclusion, this study demonstrated the positive effects of a preemptive 8 week-program of moderate continuous running on the healing of non-critical-sized defects in a rat model. Differences in healing kinetics in response to a running exercise were observed in tibia and femur. Further work should aim at investigating various types of exercises and intensities to determine the most osteogenic program. This study, however, presents the theoretical basis that MCR may be considered as a preemptive protocol to quicken bone healing in the event of bone injury, including programmed bone surgery such as knee prosthesis.

## Figures and Tables

**Table 1:** Body characteristics at 5 (T0), 15 (T8) and 19 (T12) weeks of age, and maximum aerobic speed (at 7 weeks of age, T1) in sedentary (SED) male Wistar rats and rats subjected to moderate continuous running exercise (EX).

		SED	EX	*p* Values
Weight (g)	T0	195 ± 9	207 ± 8	*p* = 0.001
	T8	469 ± 41	495 ± 43	*p* = 0.14
	T12	521 ± 50	557 ± 29	*p* = 0.08
Length (cm)	T0	19.4 ± 0.6	20.6 ± 0.7	*p* = 0.001
	T12	27.0 ± 1.0	27.5 ± 0.9	*p* = 0.35
BMI (g/cm^2^)	T0	0.52 ± 0.04	0.49 ± 0.05	*p* = 0.1
	T12	0.71 ± 0.05	0.74 ± 0.04	*p* = 0.5
MAS (m/min)	T1	23.4 ± 11.3	29.2 ± 7.9	*p* = 0.11

Groups were considered comparable at T0 based on these characteristics.

**Table 2:** Healing tissue microarchitecture and mineral density (BMD) in sedentary male Wistar rats (SED) and rats subjected to 8 weeks of moderate continuous running exercise (EX) at 1 (T9), 2 (T10), 3 (T11) and 4 (T12) weeks following non critical-sized bone defect surgery in tibias and femurs.

	Bones	Groups	Time-Points			
			T9	T10	T11	T12
BV/TV (%)	Tibia	SED	3.4 ± 1.5	8.6 ± 1.1	12.7 ± 3.3	16.3 ± 4.0 ^¶^
		EX	2.9 ± 1.6	13.4 ± 4.1 ^¥^	24.8 ± 6.8 ^¥^	34.2 ± 12.3 ^¥ ¶^
	Femur	SED	4.8 ± 0.9	10.4 ± 1.0	12.2 ± 1.8	15.9 ± 2.9 ^¶^
		EX	4.4 ± 0.9	11.1 ± 1.8	13.0 ± 2.4	22.0 ± 5.6 ^¶^
BS/BV (1/mm)	Tibia	SED	57.6 ± 11.4	47.6 ± 9.3 ^¥^	34.5 ± 6.1^¥^	21.7 ± 3.4 ^¥ ¶^
		EX	70.6 ± 26.6	43.6 ± 3.9 ^¥^	31.9 ± 8.7 ^¥^	16.4 ± 5.0 ^¥ ¶^
	Femur	SED	58.9 ± 10.7	43.0 ± 8.3 ^¥^	39.1 ± 6.8	22.8 ± 3.8 ^¥ ¶^
		EX	58.0 ± 7.4	44.3 ± 3.5 ^¥^	40.8 ± 5.2	20.3 ± 3.6 ^¥ ¶^
SMI	Tibia	SED	2.78 ± 0.47	2.38 ± 0.18 ^¥^	2.28 ± 0.24	2.01 ± 0.28 ^¥ ¶^
		EX	2.83 ± 0.15	2.40 ± 0.37 ^¥^	2.35 ± 0.56	1.97 ± 0.63 ^¥ ¶^
	Femur	SED	2.42 ± 0.21	2.43 ± 0.27	2.31 ± 0.19	2.06 ± 0.68 ^¥ ¶^
		EX	2.54 ± 0.14	2.41 ± 0.27	2.57 ± 0.17	1.76 ± 0.36 ^¥ ¶^
Tb.Pf (1/mm)	Tibia	SED	23.09 ± 9.02	12.75 ± 3.20 ^¥^	9.56 ± 2.30	5.66 ± 1.48 ^¶^
		EX	25.16 ± 6.75	12.02 ± 4.89	5.33 ± 5.50	3.59 ± 2.04 ^¶^
	Femur	SED	17.71 ± 5.55	12.46 ± 3.57 ^¥^	10.19 ± 3.37 ^¥^	4.57 ± 1.82 ^¶^
		EX	19.03 ± 3.03	12.26 ± 3.66 ^¥^	12.44 ± 1.74 ^¥^	2.98 ± 1.89 ^¶^
BMD (mg HA/cm^3^)	Tibia	SED				125.4 ± 28.
		EX				108.1 ± 11.6
	Femur	SED				107.8 ± 5.1
		EX				114.0 ± 9.2 *

Four way ANOVA (BV/TV, BS/BV, SMI and Tb.Pf) and Mann–Whitney (BMD) U tests * indicates statistically (*p* < 0.05) significant differences compared to SED group, ^¶^ indicates statistically significant differences between T12 and T9 and ^¥^ indicates statistically significant differences compared to the immediately previous time-point. *n* = 9 in each group. HA: hydroxyapatite.

**Table 3:** Plasmatic parameters in sedentary rats (SED) and rats subjected to 8 weeks of moderate continuous running exercise (EX), 4 weeks following bone defect surgery (T12).

Parameters	SED	EX
ALP (IU/L)	256 ± 113	244 ± 31
NTx (ng/mL)	52 ± 6	44 ± 6 *
PTH (pg/mL)	94 ± 86	200 ± 158

Mann–Whitney (BMD) U tests * indicates statistically (*p* < 0.05) significant differences compared to the SED group. *n* = 10 in each group.
